# Dual Drug-Loaded Nanoliposomes Encapsulating Curcumin and 5-Fluorouracil with Advanced Medicinal Applications: Self-Monitoring and Antitumor Therapy

**DOI:** 10.3390/molecules28114353

**Published:** 2023-05-25

**Authors:** Yu-Shi Liu, Jia-Wen Song, Wen-Xiao Zhong, Ming-Hao Yuan, Yu-Rou Guo, Cheng Peng, Li Guo, Yi-Ping Guo

**Affiliations:** 1 State Key Laboratory of Southwestern Chinese Medicine Resources, Chengdu University of Traditional Chinese Medicine, Chengdu 611137, China; 2 School of Pharmacy, Chengdu University of Traditional Chinese Medicine, Chengdu 611137, China

**Keywords:** curcumin, 5-Fluorouracil, liposomes, self-monitoring, cytotoxicity

## Abstract

Due to the presence of physiological barriers, it is difficult to achieve the desired therapeutic efficacy of drugs; thus, it is necessary to develop an efficient drug delivery system that enables advanced functions such as self-monitoring. Curcumin (CUR) is a naturally functional polyphenol whose effectiveness is limited by poor solubility and low bioavailability, and its natural fluorescent properties are often overlooked. Therefore, we aimed to improve the antitumor activity and drug uptake monitoring by simultaneously delivering CUR and 5-Fluorouracil (5-FU) in the form of liposomes. In this study, dual drug-loaded liposomes (FC–DP–Lip) encapsulating CUR and 5-FU were prepared by the thin-film hydration method; their physicochemical properties were characterized; and their biosafety, drug uptake distribution in vivo, and tumor cell toxicity were evaluated. The results showed that the nanoliposome FC–DP–Lip showed good morphology, stability, and drug encapsulation efficiency. It showed good biocompatibility, with no side effects on zebrafish embryonic development. In vivo uptake in zebrafish showed that FC–DP–Lip has a long circulation time and presents gastrointestinal accumulation. In addition, FC–DP–Lip was cytotoxic against a variety of cancer cells. This work showed that FC–DP–Lip nanoliposomes can enhance the toxicity of 5-FU to cancer cells, demonstrating safety and efficiency, and enabling real-time self-monitoring functions.

## 1. Introduction

With the rapid increase in cancer incidence and mortality, the prevention and treatment of malignant tumors have become major challenges to be solved in terms of global public health. Chemotherapy is currently the most commonly used treatment method, but its efficacy and selectivity are limited due to low bioavailability, drug resistance, and toxic side effects [1,2,3]. Therefore, the enhancement of other high-efficiency drugs, the development of combination chemotherapy, and treatment with active ingredients of Chinese medicine have become hotspots of modern anti-tumor research [4]. The combination of chemotherapy with natural compounds has been extensively studied to achieve synergistic efficacy and toxicity reduction through drug combinations in order to achieve a better long-term prognosis and reduce toxic side effects [5,6,7].

The expected efficacy of combinatorial administration is difficult to achieve due to the physiological barriers provided by the buccal cavity and gastrointestinal tracts, as well as physiological factors, such as liver metabolism [8]. The development of nano-drug delivery systems provides new strategies and prospects for cancer therapy, playing an important role in improving the bioavailability and activity of compounds and increasing tumor targeting [9]. One of the main purposes of nanomedicines is to reduce the dose of the drug administered by increasing bioavailability, which facilitates the reduction of adverse effects [10]. Compared to free drugs, nanocarriers may enhance permeability and retention (EPR), prolong circulation time, enable more drugs to accumulate in the tumor region, reduce drug toxicity, and improve efficacy [3]. Nanocarriers such as liposomes, magnetic nanoparticles, solid lipid nanoparticles, nano-sponges, and polymeric micelles have been developed and used as drug delivery systems [11,12]. Liposomes can simultaneously encapsulate hydrophilic and lipophilic drugs, provide site-specific targeting, and control drug release [13]. Currently, a huge number of studies are focused on the modification of the liposome structure to improve the stability of the preparation [14]. In addition, the liposomes preparation process is relatively simple, mature, and stable, displaying excellent preclinical efficacy, and it is one of the most clinically used nano-formulations [15,16]. Apart from improving therapeutic efficacy with nano-delivery systems, these systems are also crucial to achieving the real-time monitoring of drug molecules in vitro and in vivo. Although they can achieve this purpose, fluorescent markers may be potentially toxic; thus, natural fluorescent molecules are better choices [9,17].

Curcumin (CUR) is a natural polyphenolic compound isolated from the traditional Chinese medicines *Curcuma longa* L. and *C. phaeocaulis*. As the main bioactive component of *C. longa* L., it exhibits anti-tumor, anti-inflammatory, hypolipidemic, antioxidant, and antibacterial pharmacological effects [18]. It was reported to be a molecule with great potential for development into a modern drug [19]. CUR possesses high safety and is widely used in food and industry; it is also a natural molecular fluorescent agent and photosensitizer, and it can be used for the self-monitoring of drugs and photodynamic therapy [20]. However, the clinical application of CUR is severely limited by an insolubility in water, chemical instability, significant oral first-pass effect, low bioavailability, rapid metabolism in vivo, and instability regarding light and heat [21]. Co-loading curcumin with other drugs in liposomes is one of the ideal solutions [22].

The drug 5-Fluorouracil (5-FU) is an effective broad-spectrum anti-tumor drug that works by inhibiting DNA synthesis [23]. The response rate of 5-FU based on first-line treatment for advanced colorectal cancer is still 10–15%, which limits its use [15]. The drug 5-FU is prone to multidrug resistance [24], has a narrow therapeutic window, and is frequently associated with side effects, such as gastrointestinal mucositis, diarrhea, skin irritation, etc., at therapeutic doses [13,25]. Molecular compounds derived from traditional Chinese medicine are one of the viable options for combination 5-FU chemotherapy. Studies have shown that CUR can reduce the side effects of chemotherapeutic drugs [26], decrease the resistance of cancer cells to 5-FU, and improve the sensitivity of cancer cells to drugs, enhancing the killing effect on tumor cells [27,28,29,30].

In our previous study, we found that 1,2–distearoyl–sn–glycerol–3–ethanolamine phosphate–N–[methoxy (polyethylene glycol)–2000] (DSPE–MPEG 2000, DP), as a stabilizer for liposomes, can reduce the particle size, prevent the leakage of drugs, and prolong drug circulation time [14]. Therefore, in this work, DP was used as a stabilizer to prepare dual-loaded liposomes encapsulating CUR and 5-FU using the thin-film hydration method and the properties and biosafety of liposomes were investigated. More importantly, based on the fluorescence properties of CUR, the uptake of liposomes in zebrafish was monitored over 72 h, and it was verified that curcumin increases the toxicity of 5-FU in tumor cells.

## 2. Results

### 2.1. Particle Size Distribution, Zeta Potential, and Stability

**Figure 1:**
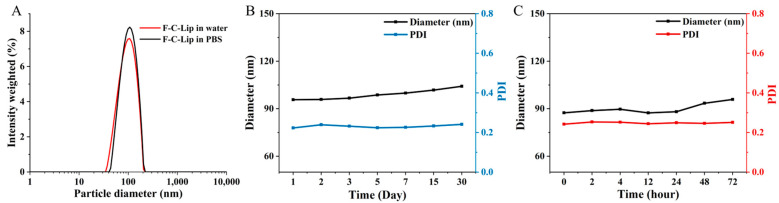
(**A**) The particle size distribution of FC–DP–Lip. (**B**) The particle size (mean value) changes of FC–DP–Lip FC–DP–Lip dispersed in water over 30 days. (**C**) The diameter (mean value) changes of FC–DP–Lip FC–DP–Lip in 10% FBS over 72 h.

The prepared liposomes were characterized by measuring particle size, zeta potential, and PDI; the results were shown in Table S1. The particle sizes of Empty-Lip in water were 64.5 ± 4.96 nm, with an acceptable PDI of 0.25. The particle size of FC–DP–Lip in water is 92.60 ± 0.88 nm, and the PDI is 0.23. The particle size distribution of FC–DP–Lip in deionized water and PBS is shown in Figure 1A, and it did not change significantly in the different solutions. Studies have pointed out that nanoparticles with particle sizes between 100 and 200 nm can effectively escape clearance by the reticuloendothelial system, have a longer circulation time than free drugs, and are more likely to accumulate in the tumor tissue due to the enhanced EPR effect [3,13]. The particle size of FC–DP–Lip was in this interval, showing the potential for long circulation and tumor tissue enrichment. FC–DP–Lip presents a negative surface charge, and the zeta potential of FC–DP–Lip was −36.0 ± 2.6 mV, while the Empty-Lip was −15.2 ± 1.1 mV. It is known that the potential size is an important factor affecting the stability of nano-drugs, and the larger the absolute value of potential, the more stable the liposome [31]. The FC–DP–Lip with DP as a stabilizer showed a small particle size, and the absolute value of the potential exceeded 20 mV, indicating that they were in a stable state. In addition, several reports suggested that negatively charged nanoparticles have the potential to prolong blood circulation time [32,33].

The particle size of FC–DP–Lip dispersed in an aqueous solution and 10% FBS demonstrated its stability, respectively (Figure 1B,C). As shown in Figure 1B, the particle size and PDI of FC–DP–Lip in water remained nearly constant over 30 days. Moreover, it remained clarified with good water dispersion and no visible precipitation, indicating that the preparation can be stored stably at 4 °C for 30 days. In addition, FC–DP–Lip can be stabilized in 10% FBS for 72 h (Figure 1C), maintaining the drug encapsulation without leakage, which was feasible for cellular experiments.

### 2.2. The TEM Image

**Figure 2:**
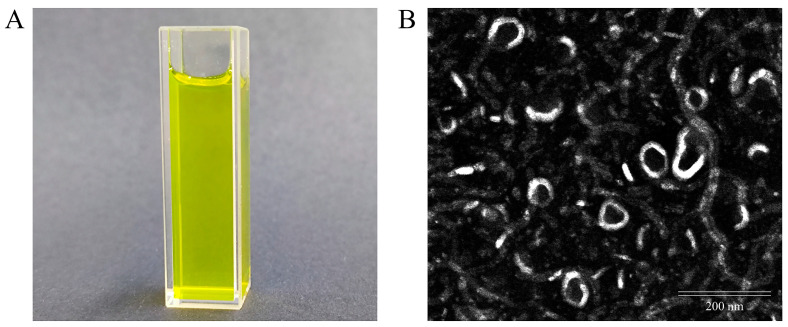
Characterization of liposomes: (**A**) optical photograph of the appearance of FC–DP–Lip; (**B**) TEM image of FC–DP–Lip.

Empty-Lip and FC–DP–Lip were successfully prepared according to the method below. FC–DP–Lip was a yellow homogeneous liquid with good dispersibility, Empty-Lip was colorless and transparent (Figure 2A and Figure S1), and the Tyndall effect was observed for both of them. As shown in the TEM images (Figure 2B), the liposome was quasi-circular, the white phospholipid bilayer of FC–DP–Lip was clearly visible, the surface was smooth, and the structure was intact. The particle size was approximately 100 nm, consistent with the results of particle size analysis, indicating that 5-FU and CUR were successfully encapsulated in the liposomes.

### 2.3. The EE% and DL% of 5-FU and CUR

**Figure 3:**
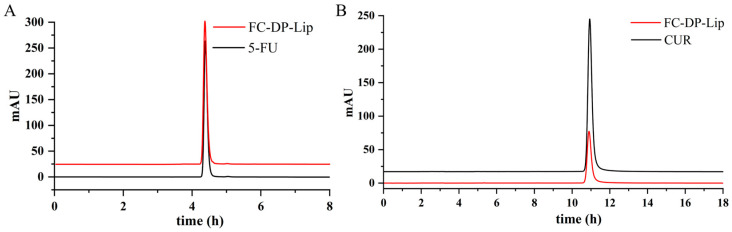
HPLC chromatogram for the determination of 5-FU (**A**) and CUR (**B**) contents in FC–DP–Lip.

The contents of 5-FU and CUR in the liposomes was measured by HPLC to calculate the EE% and DL%, and the chromatograms are shown in Figure 3. The EE% and DL% of 5-FU in FC–DP–Lip were 74.61 ± 2.30% and 8.28 ± 0.26%, while the EE% and DL% of CUR were 84.15 ± 0.30% and 1.92 ± 0.12%, respectively. The high encapsulation efficiency indicated that DP is feasible as a stabilizer for liposomes, and the FC–DP–Lip is promising for potential drug delivery applications.

### 2.4. The FTIR Spectrum, UV Absorption Spectra, and Fluorescence Emission Curve

**Figure 4:**
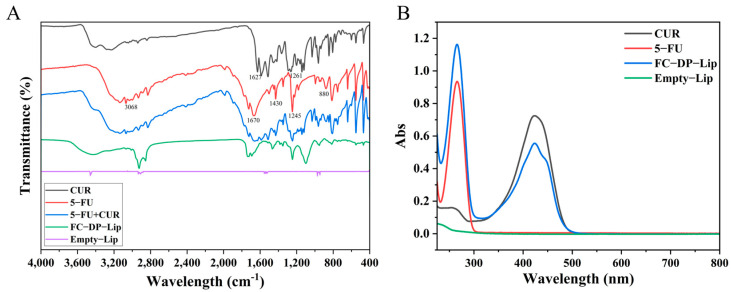
The characterization spectrum of liposomes: (**A**) the FTIR spectra of each group; (**B**) UV absorption spectra of CUR in ethanol, 5-FU, and FC–DP–Lip in water.

FTIR can verify whether the liposomes are prepared successfully. As shown in Figure 4A, many characteristic absorption peaks were shown in the CUR, with the telescopic vibrations of phenolic hydroxyl, C=O, and aromatic C–O located at 3503 cm^−1^, 1627 cm^−1^ and 1261 cm^−1^ respectively, while the olefin C–H bending vibration was located at 1429 cm^−1^, and 1025/856 cm^−1^ represented the C–O–C stretching vibration. Meanwhile, 5-FU shows characteristic peaks around 3068 cm^−1^ and 1670 cm^−1^, representing the stretching vibrations for C=C and C=O, 1430 cm^−1^ for in-plane bending vibrations of C–H in –CF–CH–, 880 cm^−1^ for out-of-plane bending vibration absorption peaks of C–H in –CF–CH–, and 1245 cm^−1^ for the stretching vibration absorption peaks of C–N. The physical mixture of 5-FU and CUR showed the superposition of the characteristic peaks of both. Interestingly, when the drug was formed into dual-loaded liposomes, most of the characteristic absorption peaks were masked, indicating the formation of FC–DP–Lip.

Similarly, the results of UV absorption spectra (Figure 4B) showed typical absorbance peaks, with the maximum absorption peaks of CUR and 5-FU located at 430 nm and 265 nm, respectively. The Empty-Lip exhibited no significant absorption peaks, while FC–DP–Lip showed two absorption bands at 430 nm and 265 nm, indicating that the drugs were co-encapsulated in the FC–DP–Lip liposomes.

As shown in Figure S2, the maximum emission wavelength was 425 nm for 5-FU and 540 nm for curcumin at a fluorescence excitation wavelength at 430 nm. The fluorescence emission curves of FC–DP–Lip showed two peaks at 425 nm and 495 nm, which were presumably caused by the reaction of 5-FU and CUR co-loading.

### 2.5. In Vivo Toxicity Test in Zebrafish

**Figure 5:**
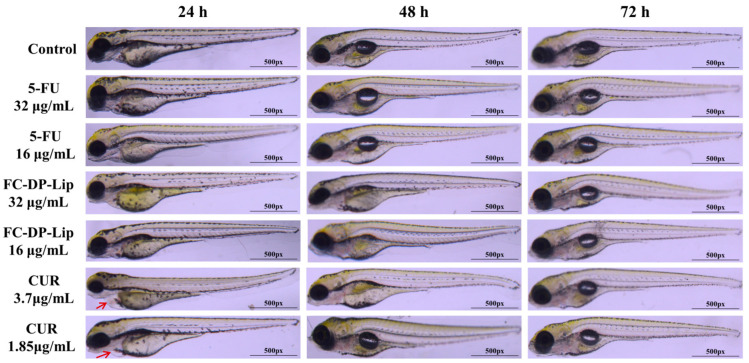
The morphology of zebrafish larvae exposed to 5-FU, FC–DP–Lip (in terms of 5-FU content), and CUR for 72 h. Pericardial edema is indicated by the red arrow.

Zebrafish (Danio rerio) is an important model aquatic organism that readily spawns under appropriate photoperiod conditions. Zebrafish embryos are optically transparent and highly sensitive to toxicant exposure [34,35,36]. Therefore, zebrafish embryos are widely used as an ideal model for toxicology studies [37]. The morphology of zebrafish embryos exposed to drugs for 72 h is shown in Figure 5. At a high dose of 32 μg/mL of 5-FU and FC–DP–Lip (in terms of 5-FU content), zebrafish developed normally, and the yolk sac was absorbed within 72 h. The zebrafish swam normally and maintained a straight posture, without deformities or mortality, which was essentially the same finding as in the control group. The same results were obtained in the low-dose group of 16 μg/mL. At high doses, the content of CUR in FC–DP–Lip was 7.5 μg/mL. As shown in Figure 5, when zebrafish were fed with CUR at 7.5 μg/mL and 3.75 μg/mL for 24 h, the zebrafish larvae exhibited slight pericardial edema and slowed movement. CUR has a short half-life and is rapidly metabolized in the body. With the prolongation of time and drug metabolism, zebrafish slowly returned to normal at 72 h. However, because of the rapid uptake of CUR, zebrafish showed partial mortality at 24–48 h. It has been reported that CUR is toxic to zebrafish embryos in a concentration-dependent manner [31], and our results confirm this finding. Our results suggested that FC–DP–Lip has excellent biocompatibility and can alleviate the irritation of high CUR concentrations in zebrafish larvae by slow drug release. Based on this, FC–DP–Lip was calculated at the CUR concentration, and a safe concentration was selected for the subsequent in vivo uptake test in zebrafish.

### 2.6. Uptake of FC–DP–Lip in Zebrafish

**Figure 6:**
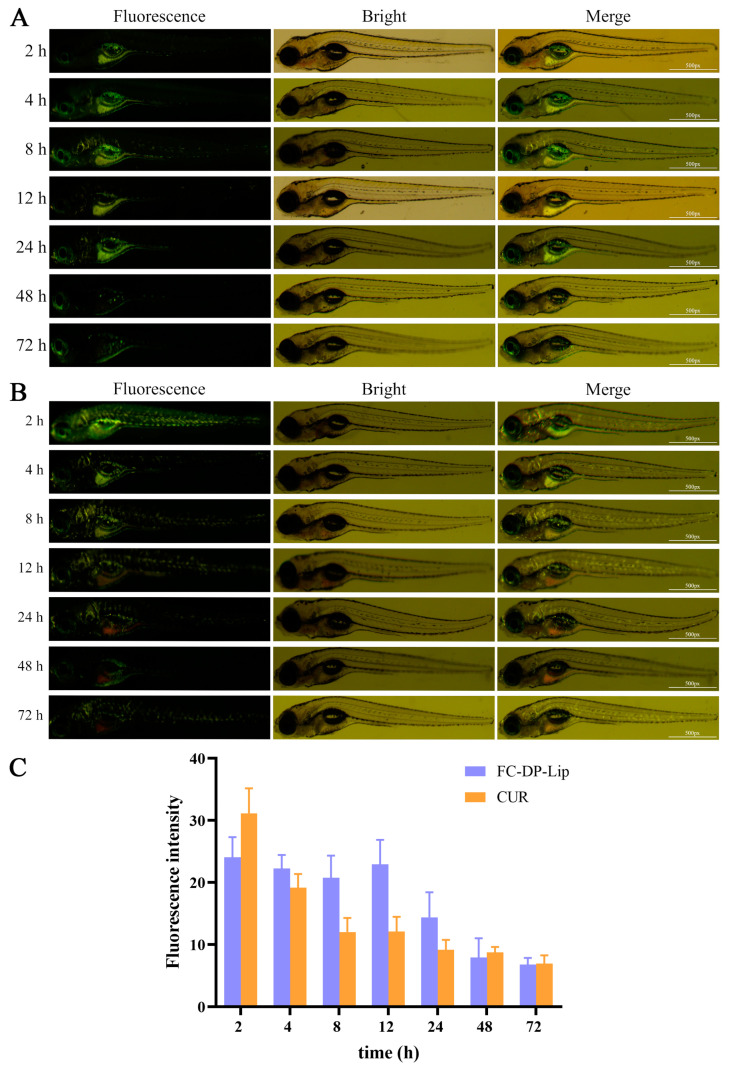
In vivo fluorescence imaging of zebrafish uptake of FC–DP–Lip (in terms of CUR content) (**A**) and CUR (**B**) at the designated time intervals. (**C**) The in vivo fluorescence intensity of zebrafish after drug ingestion was calculated with Image J software. Bars indicate means ± SD.

The zebrafish model was chosen for in vivo optical monitoring because of its convenient optical imaging. Based on the auto-fluorescence of CUR, self-monitoring and tracing can be achieved by fluorescence imaging. This quality allows for the real-time monitoring of the uptake of FC–DP–Lip and CUR by zebrafish [9]. The 5 dpf Zebrafish larvae were simply fed with 1.85 μg/mL of CUR and FC–DP–Lip (in terms of CUR content), monitored with fluorescence imaging for 72 h, and photographed at specific time intervals. As shown in Figure 6, two hours after administration, FC–DP–Lip, and CUR were rapidly absorbed by zebrafish, green fluorescence could be detected in vivo, and the signals could be detected within 72 h. In terms of drug distribution, FC–DP–Lip was mainly distributed in the gastrointestinal area of zebrafish, with less distribution in the whole body, and the fluorescence was significantly decreased after 24 h (Figure 6A). Meanwhile, CUR showed strong fluorescence within 4 h (Figure 6B). Although the drug was distributed throughout the body, it was predominantly located in the gastrointestinal tract and then gradually metabolized out of the body. From the fluorescence intensity evaluation(Figure 6C), the fluorescence intensity of CUR in zebrafish was higher than that of FC–DP–Lip at 2 h, indicating a faster initial absorption of CUR, followed by a rapid decrease in uptake. FC–DP–Lip showed a stable uptake in zebrafish, and the fluorescence intensity of FC–DP–Lip exceeded that of CUR after 4 h, gradually decaying metabolically after 24 h, but this uptake was always higher than the uptake of CUR. The fluorescence of the drug can still be monitored up to 72 h after a longer period of release and metabolism in vivo.

The real-time uptake results showed that FC–DP–Lip readily penetrated zebrafish tissues, and no mortality or deformities were observed in zebrafish within 72 h of administration, which was consistent with the biosafety evaluation results. Notably, FC–DP–Lip was mainly distributed in the gastrointestinal tract of zebrafish. The drug 5-FU is commonly used in clinically gastrointestinal tumors, and this agent has shown the potential to accumulate in the gastrointestinal tract. The formulation had a longer retention time and fluorescence intensity in vivo, demonstrating that DP, as a stabilizer, can prolong the long circulation time of the drugs and enhance the effect.

### 2.7. Cell Cytotoxicity

**Figure 7:**
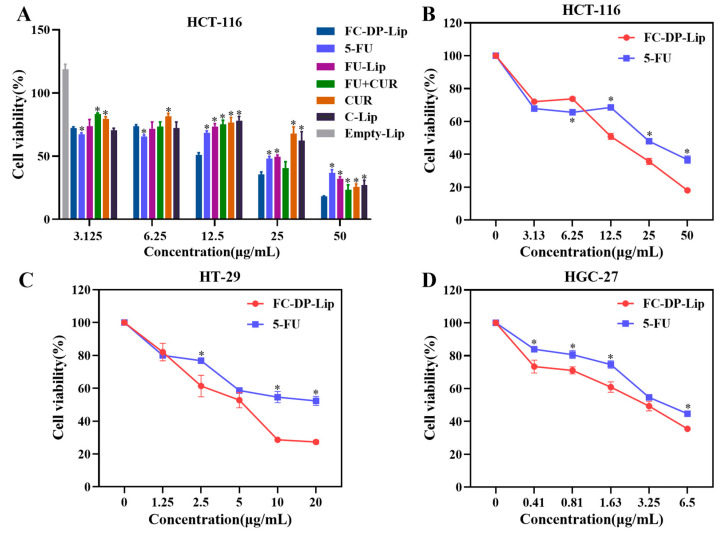
FC–DP–Lip, CUR, 5-FU and CUR + 5-FU on HCT-116 cells (**A**). In vitro cytotoxicity results of FC–DP–Lip and 5-FU on HCT-116 (**B**), HT-29 (**C**) and HGC-27 (**D**) cells. (*n* = 3). The administered concentrations were calculated based on 5-FU content, and the actual CUR concentration was 12 μg/mL. Bars indicate means ± SD, * *p* < 0.05, in comparison with the FC–DP–Lip group.

The in vitro cytotoxic effects of FC–DP–Lip and 5-FU were evaluated using HT-29, HCT-116, and HGC-27 cell lines; the results are shown in Figure 7. Liposomes loaded with a single drug, FU-Lip, and C-Lip, were prepared in the same method, and the effects of Empty-Lip, CUR, FU-Lip, and C-Lip on cell proliferation were tested with HCT-116 cells to exclude interference. As shown in Figure 7A, Empty-Lip was not cytotoxic and promoted cell proliferation, while FU and FU-Lip showed concentration-dependent cytotoxicity against HCT-116 cells. The concentration of CUR (12 μg/mL) was equivalently converted based on the drug concentration in FC–DP–Lip. In Figure 7A, the cytotoxicity with the actual CUR content as the horizontal coordinate is shown separately in Figure S3. The CUR levels in FC–DP–Lip were limited, and CUR and C-Lip exhibited cytotoxicity only at the highest concentrations. The physical mixture of 5-FU and CUR (the FU + CUR group) inhibited the proliferation of HCT-116 cells at 25–50 μg/mL. Notably, FC–DP–Lip exhibited the strongest tumor cytotoxicity, which was superior to the liposomal formulation of a single compound, and also to the physical mixture of 5-FU and CUR.

In the preparation of liposomes, the amount of CUR added was small, and its main role was to assist in enhancing the effect, as well as the fluorescent indicator and tracer; therefore, the concentration of FC–DP–Lip in the subsequent cytotoxicity experiments was calculated based on the 5-FU content. As shown in Figure 7B–D, FC–DP–Lip and 5-FU showed anti-proliferative activity against three cancer cell lines, demonstrating a wide range of antitumor effects. FC–DP–Lip exhibited stronger cytotoxic effects on HT-29, HCT-116, and HGC-27 cells and showed concentration dependence with IC50 values of 12.53, 5.03, and 2.90 μg/mL (in terms of 5-FU content), respectively. The cytotoxic effect of FC–DP–Lip was the strongest on the HGC-27 cells, followed by the HT-29 and HCT-116 cells, indicating that the drug is selective and sensitive to tumor cells. The cytotoxicity of FC–DP–Lip on gastrointestinal tumor cells was consistent with the fluorescence distribution targeting in zebrafish and also consistent with the clinical application of 5-FU in gastrointestinal tumors. Cytotoxicity results suggested that the antitumor activity of 5-FU can be enhanced by combining it with small amounts of CUR and preparing it into liposomal formulations.

## 3. Discussion

Fluorescence monitoring and tracing techniques are indicative in the pre-development phase of drugs, and it is highly desirable to achieve real-time monitoring of the uptake or distribution of drug molecules in vitro and in vivo, while improving efficacy. So far, a prevalent strategy to achieve monitoring is to encapsulate chemotherapeutic drugs in fluorescent nanocarrier materials. It is also practicable to construct nanoparticles with drugs and fluorescent markers [9,17]. However, nanocarriers and fluorescent markers may have potential toxicity, and the low drug loading caused by them limits the application of multifunctional therapy. Therefore, natural fluorescent small molecules with good pharmacodynamic activity are highly desired for drug development. CUR has been reported to be the star molecule with the potential to be developed into a modern drug with multiple pharmacological activities. It is also a natural molecular fluorescent agent and photosensitizer for the self-monitoring of drugs [20].

Other systems with dual-loaded 5-FU and curcumin nanoparticles or liquid nanostructures have also been constructed. Nano-drug delivery systems can increase drug loading capacity, as well as the targeted and controlled release of 5-FU and CUR [38]. The combination of CUR and 5-FU has been shown to enhance antitumor effects and reduce 5-FU resistance and side effects, but less attention has been paid to the fluorescent tracer effect of CUR. Previous studies have found that curcumin is slowly released in nano drugs, and drug distribution can be monitored by fluorescence imaging. The autofluorescence of curcumin avoids the use of fluorescent markers or fluorescent nanocarriers, contributing to improved drug loading and security. In this study, CUR was co-encapsulated with 5-FU in liposomes. Applying the fluorescence of CUR, the uptake and distribution of nanoliposomes in zebrafish can be monitored in real-time, which has positive implications for the exploration of drug effectiveness and in vivo retention time. Our findings increase the focus on the autofluorescence of natural compounds and contribute to the development of potential natural drugs.

High doses of curcumin exhibit cytotoxicity, but its poor solubility and low bioavailability limit its application in the medical field. Nanoliposomes can significantly improve the solubility and bioavailability of CUR, but they still exhibit poor stability and self-leakage in regards to drugs with specific physical and chemical properties. The drug 5-FU is water-soluble, and the encapsulation and high 5-FU loading is one of the difficult points in the preparation of liposomes. The addition of stabilizers is a viable solution. The effects of different stabilizers such as DP, VE-TPGS, and PLGA were compared, and DP was found to be the best choice. The addition of DP could better prevent drug leakage, significantly improve the EE and DL of curcumin, and prolong the stabilization period of the liposomes. The prepared FC–DP–Lip was morphologically intact, with a clear phospholipid bilayer, homogeneous particle size, and stable solution. High doses of CUR induced pericardial edema in zebrafish, which had a slight effect on growth and development, while FC–DP–Lip eliminated the adverse effects and was relatively safe. The addition of CUR enhanced the activity of 5-FU, and FC–DP–Lip exhibited better antitumor activity.

## 4. Materials and Methods

### 4.1. Materials

Curcumin (purity > 98%) was purchased from Push Bio-Technology Co., Ltd. (Chengdu, China). The drug 5-FU (purity > 98%) was purchased from Macklin Biochemical Co., Ltd. (Shanghai, China). Soybean lecithin, cholesterol, and DSPE–MPEG 2000 were purchased from A. V. T. (Shanghai, China) Pharmaceutical Co., Ltd. Methanol and acetonitrile of high-performance liquid chromatography (HPLC) grade were purchased from Thermo Fisher Scientific (Shanghai, China). HPLC-grade phosphoric acid and acetic acid were purchased from Chengdu Kelong Chemical Co., Ltd. (Chengdu, China). HQ fetal bovine serum (FBS) was purchased from TransGen Biotech Co., Ltd. (Beijing, China). DMEM and McCoy’s 5A culture medium were purchased from HyClone™ (Beijing, China). Cell Counting Kit-8 (CCK-8) was purchased from Labgic Technology Co., Ltd. (Beijing, China). Phosphate-buffered saline (PBS; pH 7.4) was prepared in the laboratory, and ultrapure water was produced using a ULUPURE integral water purification system (Chengdu, China). All other chemicals were of analytical grade.

### 4.2. Animals and Cells

Wild-type AB line zebrafish were supported by the zebrafish experimental platform at Chengdu University of Traditional Chinese Medicine (Chengdu, China). Human colorectal cancer cell HCT-116, HT-29, and human gastric cancer cell HGC-27 cell lines were provided by the State Key Laboratory of Southwestern Chinese Medicine Resources.

### 4.3. Preparation of Liposomes

Liposomes encapsulating 5-FU and CUR were prepared by the thin-film hydration method. Briefly, cholesterol/soybean lecithin (mass ratio 1:4), CUR 2.0 mg, and the appropriate amount of DP were placed in a roun-bottom flask and dissolved in methanol/chloroform (*v*/*v* 1:2), and the organic solvent was slowly removed on a rotary evaporator to form a yellow, uniform film. Then, the thin lipid film was hydrated with 10.0 mL UP water containing 5-FU (8.0 mg) by shaking it in a water bath for 30 min; the resulting liposomes were ultra-sonicated (300 W) for 3 min to obtain homogeneous-sized liposomes. The liposomal liquid was further centrifuged at 10,000 rpm for 10 min to remove the free drug and then extruded and filtered. The resulting liposomes co-loaded with 5-FU and CUR were labeled as FC–DP–Lip.

### 4.4. Particle Size and Morphology Determination

The particle size, polydispersity index (PDI), and zeta potential of the liposomes were determined by a particle analyzer (Litesizer 500, Anton Paar, Graz, Austria). Before measurement, samples were diluted with UP water to the same multiple and measured at room temperature. The experiment was repeated three times. The appearance and morphology of the FC–DP–Lip were evaluated by transmission electron microscopy (TEM). The samples were placed on a copper net, negatively stained with 1% (*w*/*v*) phosphotungstic acid, and then observed and photographed.

### 4.5. Determination of Encapsulation Efficiency (EE%) and Drug Loading (DL%)

The EE% and DL% of FC–DP–Lip were analyzed by HPLC (Agilent, 1260, Santa Clara, CA, USA). After centrifugation of the liposomes, the suspensions of the encapsulated drug were collected, dispersed with methanol, and sonicated for 10 min to release the entrapped drugs, i.e., 5-FU and CUR. The analysis was performed on a C_18_ analytical column (4.6 mm × 150 mm, 5 μm), and the column temperature was set at 30 °C, the injection volume was 10 μL, and the flow rate was 1.0 mL/min. The mobile phase for detecting 5-FU was 95% acidified water (pH 3.5, adjusted with phosphoric acid) and 5% methanol, and the detection wavelength was 265 nm. The mobile phase for CUR was 52% 0.4% acetic acid and 48% acetonitrile at 430 nm. The EE% and DL% are calculated according to the following formula:

(1) $$\mathrm{EE}\%=\frac{\mathrm{The}\;\mathrm{weight}\;\mathrm{of}\;\mathrm{drug}\;\mathrm{encapsulated}}{\mathrm{The}\;\mathrm{weight}\;\mathrm{of}\;\mathrm{the}\;\mathrm{drug}\;\mathrm{added}}\times 100\%$$

(2) $$\mathrm{DL}\%=\frac{\mathrm{The}\;\mathrm{weight}\;\mathrm{of}\;\mathrm{the}\;\mathrm{drug}\;\mathrm{measured}}{\mathrm{The}\;\mathrm{weight}\;\mathrm{of}\;\mathrm{all}\;\mathrm{materials}}\times 100\%$$

### 4.6. Stability Study of Liposomes

The storage stability of FC–DP–Lip over 30 days was evaluated to explore the rationality of the preparation process. The samples were diluted in deionized water, PBS, and 10% FBS, respectively, for analysis of particle size and PDI.

### 4.7. Characterization Determination

To verify the successful preparation of liposomes, the mid-infrared spectrum of the liposomes was measured by FTIR (PerkinElmer, Waltham, MA, USA). The samples were prepared by the potassium bromide pressing method, and the scanning wavelengths were 4000 to 400 cm^−1^. Meanwhile, the sample solutions were scanned by UV spectroscopy (Shimadzu, Kyoto, Japan) at 800~200 nm. The fluorescence emission curves of FC–DP–Lip, 5-FU, and CUR at 300–700 nm were scanned with an enzyme standardizer (Flash Shanpu, Shanghai, China) at a fixed excitation wavelength of 430 nm.

### 4.8. Toxicity Evaluation in Zebrafish

Three-month-old AB line zebrafish were maintained and raised under standard conditions, as described previously [36]. Adult zebrafish and embryos were cultured at 28 °C in a 14 h light/10 h dark cycle with culture water at a pH of 7.2–7.5 and a conductivity of 500–550 μS/cm. All experiments were conducted following legal regulations, with ethical approval from the Institutional Animal Ethics Committee of the Chengdu University of TCM. Embryos with 48 h post fertilization (hpf) were randomly selected and incubated with high and low doses of drugs (FC–DP–Lip, 5-FU, CUR) for 72 h, observed, and photographed with a Leica fluorescence microscope.

### 4.9. Uptake of Liposomes in Zebrafish

The autofluorescence of CUR can be used to evaluate the uptake of liposomes in zebrafish. Zebrafish embryos develop rapidly, and larval organs mature in about 5 days, which is convenient for monitoring drug uptake and distribution. Therefore, 5 days post fertilization (dpf), healthy larvae were randomly selected and incubated with 1.85 μg/mL CUR and FC–DP–Lip (in terms of CUR content), respectively. The fluorescence in the larvae was observed by fluorescence microscopy and photographed immediately at designated time intervals after drug administration. All images in the different groups were obtained with the same parameters (exposure time, ISO, and aperture) for comparative studies. The fluorescence intensity and area, respectively, were calculated by Image J software.

### 4.10. Cell Cytotoxicity Assay

Colon cancer cells HCT-116 and HT-29 were incubated in McCoy’s 5A medium containing 10% FBS and 1% penicillin-streptomycin at 37 °C in a humidified atmosphere containing 5% CO_2_, while gastric cancer cells HGC-27 were incubated in DMEM with 10% FBS and 1% penicillin-streptomycin. The target cells in the logarithmic growth period were inoculated into 96-well plates at a density of 4000 cells/100 μL medium. After incubation for 24 h, the medium was replaced with fresh medium (with 1% FBS and 1% penicillin-streptomycin) containing 5-FU and FC–DP–Lip at different concentrations (0.41–50 μg/mL) for 48 h. Then,10 μL CCK-8 reagent was added and incubated in the dark at 37 °C for 1 h. Finally, absorbance was measured by a microplate reader (Gen5, BioTek) at 450 nm, and the cells incubated with a blank medium were used as the control group. The experiment was performed in triplicate. The cell viability was calculated using the formula below:

(3) $$\mathrm{Cell}\;\mathrm{viability}\;\left(\%\right)=\frac{\mathrm{OD}\;\mathrm{experimental}}{\mathrm{OD}\;\mathrm{control}}\times 100\%$$

## 5. Conclusions

In summary, dual drug-loaded liposomes encapsulating CUR and 5-FU were successfully constructed by using DP as a stabilizer. FC–DP–Lip, as an efficient and safe drug delivery system, shows good biocompatibility. In addition, these nanoliposomes do not affect zebrafish embryonic development, but enhance the cytotoxic effect of 5-FU on tumor cells. Based on the fluorescence of CUR, zebrafish fluorescence imaging showed that FC–DP–Lip could be used as a real-time self-monitoring nano-drug for tumor therapy. This nanoliposome drug delivery system based on the auto-fluorescent drugs CUR and 5-FU may open new potential areas for combination therapy and the monitoring of cancer.

## Data Availability

The data presented in this study are available upon request from the corresponding author.

## Supplementary Materials

The following supporting information can be downloaded at: https://www.mdpi.com/article/10.3390/molecules28114353/s1, Figure S1: Optical photograph of the appearance of Empty-Lip and FC–DP–Lip, Figure S2: Fluorescence emission spectra of 5-FU, CUR and FC–DP–Lip, Figure S3: In vitro cytotoxicity results of CUR, C-Lip and 5-FU on HCT-116; Table S1: The particle size, zeta potential and PDI results of liposomes, Table S2: The IC_50_ values of FC–DP–Lip and 5-FU in HT-29, HCT-116 and HGC-27 cell lines in vitro (μg/mL).

## Sample Availability

Samples of the compounds are not available from the authors.
