# Supplementary material for: Dual Drug-Loaded Nanoliposomes Encapsulating Curcumin and 5-Fluorouracil with Advanced Medicinal Applications: Self-Monitoring and Antitumor Therapy
Source: Molecules. 2023 May 25;28(11):4353. doi: 10.3390/molecules28114353 (PMC10254180; doi:10.3390/molecules28114353)
Supplement: Supplementary file 1 [file molecules-28-04353-s001.zip › supplementary 5-FU and CUR.pdf]

Table S1. The particle size, zeta potential and PDI results of liposomes

	particle size (nm)	PDI (%)	zeta potential (mV)
Empty-Lip	64.5 ± 4.96	0.25	-15.2±1.1
FC-DP-Lip	92.60±0.88	0.23	-36.0±2.6

Table S2. The IC_{50} values of FC-DP-Lip and 5-FU in HT-29, HCT-116 and HGC-27 cell lines in vitro (μg/mL)

	HT-29	HCT-116	HGC-27
FC-DP-Lip	5.03	12.53	2.90
5-FU	17.42	23.73	5.01

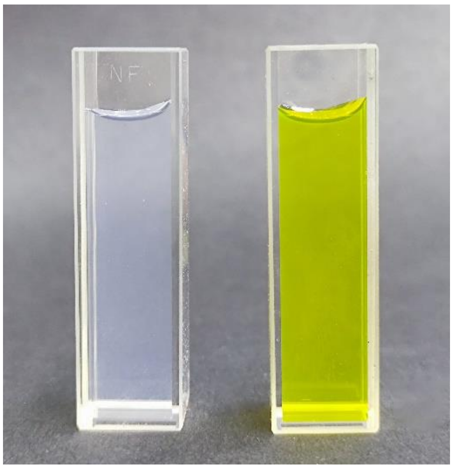

Figure S1. Optical photograph of the appearance of Empty-Lip (left) and FC-DP-Lip (right).

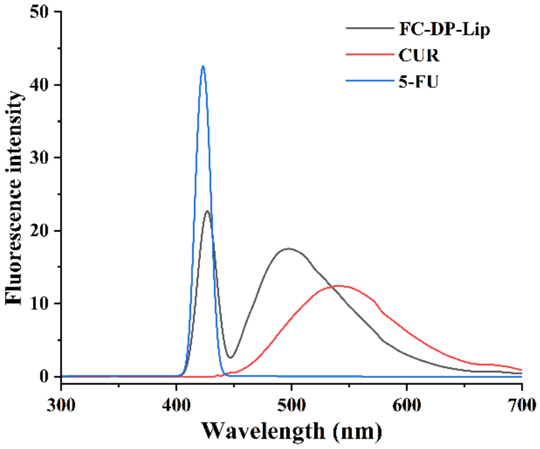

Figure S2. Fluorescence emission spectra of 5-FU, CUR and FC-DP-Lip

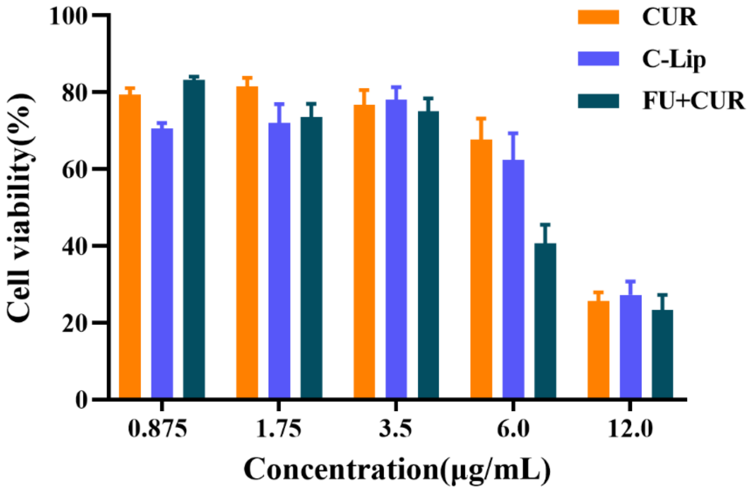

Figure S3. In vitro cytotoxicity results of CUR, C-Lip and 5-FU on HCT-116 (n=3). The administered concentrations were calculated based on CUR content. Bar indicate means ± SD.
